# Construction Notes

**Published:** 1894-12-01

**Authors:** 


### CONSTRUCTION NOTES.

**New Cottage Hospital for the Rhondda.**

The ceremony of laying the memorial stone of a cottage hospital for Porth and district in the Rhondda was performed quite recently. The hospital is being erected on Cemetery Road, on an elevated position overlooking the Rhondda Valley. The building consists of two wards, each containing four beds, matron's room, doctor's room, nurse's day room, operating room, kitchen, scullery, and offices on the ground floor, whilst private wards and bed rooms are placed on the first floor. The building is to be of local red brick, dressings being provided for the frontage, the remainder of the external wards being cemented. Provision has been made for future extension, if required, to the extent of eight beds. The contract has been carried out by Messrs. Charles Jenkins and Sons, of Porth, at an estimate of £2,500, the builders working from the designs of Mr. F. Gibson, architect. The site is the gift of Colonel Turberville, and the cost of the building is defrayed by public subscription.

**New Hospital and Dispensary at Retford.**

By the establishment of the above institute the district will be provided with a thoroughly well-equipped hospital and dispensary. The cottage hospital, which it has succeeded, having proved itself insufficient to meet the ever-increasing demands on its accommodation, it was decided to extend the premises, and a private house and grounds have been secured by the promoters for this object. This newly-acquired building necessarily required adaptation to the new office it was to serve, and during the past month or two the interior has undergone considerable changes and the plan of the rooms altered to suit their future purpose. Mr. John Wilson, of Retford, has had all this work in hand, and has carried it out in a most efficient manner. The house and grounds include considerably over one and a half acres, and there are ten principal rooms with a proportionate number of accessory departments, kitchen sculleries, pantries, &c. On either side of the entrance hall are the two main wards, male and female. Each is 26 feet long by 17, and they are admirably adapted for the use to which they are put, being lofty and well ventilated. In the male ward at present are three beds, in the female five, and a cot. Electric bells are fitted to each bed. In each, in addition to the entrance door, is another leading to the bath-room and ante-room, and behind the main wards, on the left side, are the kitchens and sculleries, on the right the matron's room. The only absolutely new extension is a small wing comprising operation and bath-room, &c. The rooms on the first floor remain practically unaltered for the present. The lodge at the entrance gate is to be used as the dispensary, it contains four good rooms. In the grounds are a stable and coach-house, and near to the main building a laundry and a mortuary. The formal opening of this institution took place quite recently.

